# Nose-to-brain selective drug delivery to glioma via ferritin-based nanovectors reduces tumor growth and improves survival rate

**DOI:** 10.1038/s41419-024-06653-2

**Published:** 2024-04-13

**Authors:** Francesco Marrocco, Elisabetta Falvo, Luciana Mosca, Giada Tisci, Alessandro Arcovito, Alice Reccagni, Cristina Limatola, Roberta Bernardini, Pierpaolo Ceci, Giuseppina D’Alessandro, Gianni Colotti

**Affiliations:** 1 Department of Physiology and Pharmacology, Sapienza University, Rome, Italy; 2 Institute of Molecular Biology and Pathology, Italian National Research Council IBPM-CNR, Rome, Italy; 3 Department of Biochemical Sciences, Sapienza University, Rome, Italy; 4 Dipartimento di Scienze Biotecnologiche di Base, Cliniche Intensivologiche e Perioperatorie, Università Cattolica del Sacro Cuore, Largo F. Vito 1, 00168 Rome, Italy; 5 Fondazione Policlinico Universitario “A. Gemelli”, IRCCS, Largo Agostino Gemelli 8, 00168 Rome, Italy; 6 Department of Physiology and Pharmacology, Sapienza University, Laboratory affiliated to Institute 17 Pasteur Italia, Rome, Italy; 7 IRCCS Neuromed, Pozzilli, IS Italy; 8 Dipartimento di Scienze Cliniche e Medicina Traslazionale Università degli Studi di Roma “Tor Vergata”, Rome, Italy; 9 Thena Biotech, Latina, Italy

**Keywords:** Biochemistry, Cell delivery, CNS cancer

## Abstract

Gliomas are among the most fatal tumors, and the available therapeutic options are very limited. Additionally, the blood-brain barrier (BBB) prevents most drugs from entering the brain. We designed and produced a ferritin-based stimuli-sensitive nanocarrier with high biocompatibility and water solubility. It can incorporate high amounts of the potent topoisomerase 1 inhibitor Genz-644282. Here, we show that this nanocarrier, named The-0504, can cross the BBB and specifically deliver the payload to gliomas that express high amounts of the ferritin/transferrin receptor TfR1 (CD71). Intranasal or intravenous administration of The-0504 both reduce tumor growth and improve the survival rate of glioma-bearing mice. However, nose-to-brain administration is a simpler and less invasive route that may spare most of the healthy tissues compared to intravenous injections. For this reason, the data reported here could pave the way towards a new, safe, and direct ferritin-based drug delivery method for brain diseases, especially brain tumors.

## Introduction

Gliomas are a family of tumors whose cells of origin may be neural stem cells (NSCs), NSC-derived astrocytes, and oligodendrocyte precursor cells (OPCs). They account for about 80% of all malignant brain tumors in the central nervous system (CNS) [1–3]. Treatment and prognosis of gliomas depend on the glioma type and on several prognostic factors [4]. However, despite recent advances, treatment of gliomas is often problematic and ineffective. The survival of high-grade glioma patients is less than 15 months even after surgery, and the overall 5-year survival rate is below 7% [2, 5]. The current standard of care for high-grade gliomas consists of maximal safe resection of the tumor followed by chemoradiation therapy using the alkylating agent temozolomide [6]. Several bottlenecks affect the treatment of glioma. The removal of the tumor is hampered by the infiltrative nature of most gliomas, and by the difficult and unreliable identification of the tumor margins [7]; radiotherapy has efficacy concerns, in addition to acute, early-delayed and late-delayed neurological side effect induced in CNS [8]. Bevacizumab (Avastin) and temozolomide are the most used drugs against high-grade gliomas. They show a good safety profile and demonstrated some clinical benefit, although only little prolonged progression-free survival or overall survival improvement could be demonstrated [6, 9, 10]. Further, high-grade gliomas inevitably recur, because they are often inherently resistant to therapy, their genetic heterogeneity hampers targeting of single oncogenic pathway, and they are in a microenvironment that includes microglia that can be tumor-supportive and that influences responses to therapy [11–15].

In addition, the blood-brain barrier (BBB) is an obstacle that almost all chemotherapeutic drugs need to overcome. The BBB is a complex, extensive, multicellular protective cell barrier between the CNS and the peripheral blood circulation. It consists of brain capillary endothelial cells, a basement membrane, pericytes, astrocytes, and tight junctions. The BBB maintains CNS homeostasis by tightly controlling the passage of specific nutrients, such as amino acids, glucose, nucleosides, and fatty acids, and restricting the passage of harmful xenobiotic molecules, such as neurotoxic agents, from the vasculature into the extracellular fluid of the CNS. The BBB prevents almost all macromolecules and at least 98% of small-molecule drugs, including most chemotherapeutics, from entering the brain. Therefore, drug delivery strategies aimed at significantly improving BBB crossing and drug accumulation in gliomas are highly valuable against brain tumors and diseases [16–18].

Nanocarriers show promising clinical potential for glioma targeting. Most nanocarriers studied for brain delivery are surface-modified to exploit cellular receptor-, transporter-, or adsorption-mediated mechanisms for drug delivery. The receptor-mediated transcytosis strategy is the most promising one, as it employs highly expressed endocytosis-related receptors in BBB endothelial cells, such as transferrin receptors (TfRs), insulin receptor, lactoferrin receptor, low-density lipoprotein receptors, folate receptor, melanotransferrin, CD98, and a variety of antibodies and ligands [16, 18–23]. Nanocarriers decorated with appropriate ligands could thus bind these receptors and cross the BBB via receptor-mediated transcytosis.

TfRs are the most studied and validated target protein for brain delivery approaches, and many technologies targeting TfRs have been used, mostly using antibodies [18]. The ferritin/transferrin receptor TfR1 (CD71) is expressed in normal cells such as erythrocytes, hepatocytes, and intestinal cells, where it is needed for iron metabolism, since it binds specifically and with high affinity transferrin (Tf) and the H subunit of ferritin and allows internalization of these iron-binding proteins [24–28]. In energy-requiring cells, such as brain endothelial cells, and in rapidly proliferating cells such as cancer cells, the expression level of TfR1 is significantly elevated; the expression of TfR1 on the surface of many types of cancer cells is up to 100-fold higher than that of normal cells [29, 30]. The high expression of TfR1 in both glioma cells and brain endothelial cells makes TfR1 an ideal target receptor for drug delivery across the BBB and to the glioma. Due to the high concentration of endogenous Tf in the bloodstream, competition may occur with Tf-modified nanocarriers, that often exhibit unsatisfying targeting ability and safety [23].

Ferritin (Ft) is a spherical nanocage composed of 24 subunits (heavy (H) or light (L)), able to reversibly assemble and disassemble in a pH-dependent fashion, with a hollow center for storing up to 4500 iron (Fe^3+^) ions [31, 32]. Ft-based nanocarriers have been developed, mostly using their cavity to incorporate drugs and other payloads, in order to deliver them to tumors. Ferritin can be produced with high purity and is inexpensive, non-immunogenic, biodegradable, highly soluble and able to protect the payload from reducing agents and from metabolism [33], and is able to pass the BBB via TfR1-dependent transcytosis [24, 34]. However, the delivery of drugs into the CNS using Ft-based nanocarriers is less investigated, with only a few studies using doxorubicin- and paclitaxel-loaded ferritin for glioma treatment [24, 35–37]. All of these studies were performed using the H chain of Ft (HFt), the only one specifically able to bind to TfR1. Notably, HFt binds to a different epitope than Tf, so its binding (and therefore, its ability to cross the BBB and enter glioma cells) cannot be competitively inhibited by endogenous Tf [26, 28].

**Fig. 1:**
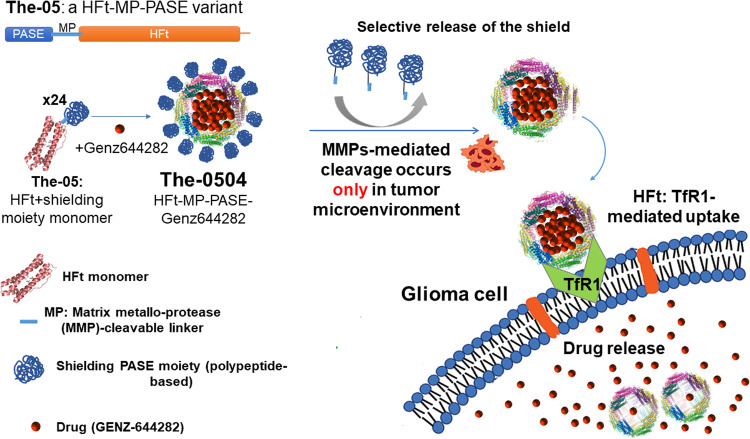
Mechanism of action of The-0504. The-05 is composed by polypeptide comprises the N-terminal shielding PASE moiety (blue), a matrix metalloprotease (MMP)-cleavable linker (clear blue) and a C-terminal human ferritin heavy chain (HFt, orange), that fold into a The-05 monomer. In the presence of the drug Genz-644282, using a simple pH-dependent association-dissociation protocol, the monomer assembles into a The-0504 24-mer, where the drug is encapsulated into the stimuli-sensitive protein, that can be administered to the tumor mice model. In the tumor microenvironment MMP-mediated cleavage of the PASE shield occurs, the The-0504 is internalized by glioma cells via TfR1, and the drug is released into glioma cells, where it inhibits topoisomerase I.

We therefore designed a stimuli-sensitive HFt-based nanocarrier, in which HFt is joined with a peptide sequence specifically cleavable by metalloproteases 2 and 9 (MP) to a shielding polypeptide named PASE, which is composed of only proline (P), alanine (A), serine (S), and glutamic acid (E) (Fig. 1). The PASE polypeptide increases HFt water solubility, causes a tenfold decrease in TfR binding affinity, and extends the half-life of the nanocarrier in the bloodstream compared to native HFt [38]. At the tumor site, the PASE-linked MP sequence is selectively cleaved off by matrix metalloproteases 2 and 9 of the tumor microenvironment, leading to HFt unmasking, and hence to binding to TfR1, selective internalization in the cancer cells, and tumor killing. The HFt-MP-PASE protein (also called The-05) incorporating doxorubicin, mitoxantrone and the potent non-camptothecin topoisomerase I inhibitor Genz-644282 was proved to be efficacious against several tumor models of different origin and well-tolerated in multiple-cycle repeat-dose study in both mice and rats [25, 38–40].

The compound formed by The-05 and Genz-644282 is named The-0504, and here we evaluated its activity on murine glioma cells, both in vitro and in in vivo model. In order to be able to use our stimuli-sensitive delivery system, first we measured the levels of TfR1 in these glioma cells compared to normal brain cells. In addition, the affinity between HFt and both human and mouse TfR1 were evaluated by Surface Plasmon Resonance experiments.

Then, we tested the ability of our nanovector system to reduce glioma growth and increase overall survival. To this end, we evaluated two different administration routes: intravenous and intranasal. The nose-to-brain route has several advantages, since it is a highly accessible, non-invasive pathway of delivery, characterized by rapid onset of action and high cerebral bioavailability, that has been tested in many investigational studies in the past five years and has the potential to pave the way for a brighter future in the management of brain diseases; a few approved nasal formulations exist for brain disorders such as opioid overdose, migraine, depression, pain and anxiety [16]. The nose-to-brain route is easily accessible, highly vascularized, and physically short. It is directly connected to the brain via the olfactory pathway, the respiratory pathway, the systemic pathway, and the nasopharynx-associated lymphoid tissue. Importantly, it partially bypasses the BBB [16, 41]. However, intranasal administration has limitations, as the limited administration volume, mucus layer, and the possibility that drugs can be degraded and/or fail to reach the site of action. This has led to many studies on the implementation of nanoparticle systems, based on many different carriers such as liposomes, micelles and polymeric nanoparticles, to overcome these limitations and accurately deliver drugs to the site of action [16]. For these reasons, we decided to preliminarily investigate the possible use of The-0504 in brain tumors via the nose-to-brain route using an orthotopic model of murine glioma.

## Materials and methods

### Materials

Cell culture medium (Dulbecco’s modified minimum essential medium, DMEM), fetal bovine serum (FBS), penicillin G, streptomycin, glutamine, amphotericin B, sodium pyruvate, goat serum and Hoechst were from GIBCO Invitrogen (Carlsbad, CA, USA); 3-(4,5-dimethylthiazol-2-yl)-2,5-diphenyltetrazolium bromide (MTT) salt, dimethyl sulfoxide (DMSO), phosphate-buffered saline (PBS) tablet, Dulbecco’s phosphate-buffered saline, hematoxylin, eosin were from Sigma-Aldrich (Milan, Italy); paraformaldehyde solution 4% in PBS (PFA) and secondary antibodies were provided by Santa Cruz Biotechnology.

### Protein production and drug encapsulation

The production and full characterization of The-0504 is reported in [38]. The-0504 was provided as lyophilized powder by Thena Biotech (Latina, Italy).

### Animals and cell lines

Experiments described in the present work were approved by the local animal welfare body and by the Italian Ministry of Health (authorization No. 231/2015PR) in accordance with the guidelines on the ethical use of animals from the European Community Council Directive of September 22, 2010 (2010/63/EU) and the Italian D. Leg. 26/2014. The study was conducted in accordance with the ARRIVE guidelines [42].

All possible efforts were made to minimize animal suffering and to reduce the number of animals used per condition by calculating the necessary sample size before performing the experiments. Eight-weeks-old C57BL/6 N mice were obtained from Charles River (Calco, Italy) and were randomly assigned to experimental groups. The microbiological status of the animals and facility were continuously monitored.

Murine GL261 glioma cell line (kindly provided by Prof. Michela Matteoli Humanitas, Milan, Italy) and human U87MG cell line were cultured in DMEM supplemented with 20% and 10% heat-inactivated FBS respectively. In the cell culture medium 100 IU/ml penicillin G, 100 µg/ml streptomycin, 2.5 µg/ml amphotericin B, 2 mM glutamine and 1 mM sodium pyruvate were present as well. Cells were grown at 37 °C in a 5% CO_2_-humidified atmosphere and sub-cultivated when confluent.

### Surface Plasmon Resonance experiments

Surface Plasmon Resonance (SPR) experiments were carried out to assess the thermodynamical and kinetic parameters of the interaction between HFt (analyte) and human or murine TfR1 (ligands) in a Biacore X-100 apparatus. For capturing, recombinant human or murine His-tagged TfR1 stocks were 200 µg/ml. The ligands were diluted to 50 µg/ml in PBS and injected on NTA sensorchips, loaded with 0.1 M NiSO_4_. The experiments were carried out in PBS buffer + 0.005% Tween 20. Sensorgrams show HFt injection (time 0–180 s) at 30 µL/min at the following concentrations: 2, 1, 0.5, 0.25, 0.125 µM, followed by buffer injections (180–780 s) to monitor analyte dissociation. All curves were fitted as single 1:1 interactions.

For regeneration, we used 10 mM HEPES at pH 8.3 in the presence of 0.35 M EDTA according to manufacturer’s suggestion.

### MTT cell viability assay

GL261 cells and U87 MG cells were seeded into 96-well plates (10^4^ cells/well) and exposed to different concentrations of HFt-MP-PASE-Genz-644282 (1–10–100 nM in Genz-644282) to assess the viability of cells. Cell viability was determined by MTT assay. MTT solution (500 µg/ml) was added to the cells in each well and the plates were incubated for 1.5 h at 37 °C in a 5% CO_2_ incubator. The resulting formazan crystals were dissolved by adding DMSO. Optical densities (OD) were measured at 570 nm using Tecan Infinite F Nano+ plate reader and processed using Tecan iControl 2.0 software. Cell viability data was expressed relative to the variation in absorbance over time, by plotting the means of OD value measured minus reference (600 nm) for each condition versus time.

### Mice housing and treatment

Mice were housed (two or three per cage) in standard breeding cages at a constant temperature (22 ± 1 °C) and relative humidity (50%), under a 12 h-light cycle, with standard chow ad libitum. The bedding materials were changed once a week. After one week from tumor cell inoculation, the animals were treated every two days for 2 weeks (six times) intranasally with 5 or 15 µL of HFt-MP-Genz-644282 (The-0504) at a dose of 0.3 mg/kg or 0.9 mg/kg (expressed in Genz-644282) or with the same content of empty The-05 Ferritin (HFt) vector, as control. For survival experiments treatment was prolonged until sacrifice, as shown. For intravenous treatment, animals were injected with 200 µL of The-0504 at a dose of 2.0 mg/kg.

### Orthotopic GL261 cell injection

Mice were anesthetized with Rompun 20 mg/ml (75 mg/kg i.p.) + Zoletil 50/50 mg/ml (20 mg/kg i.p.) and placed in a stereotaxic head frame. Animals were stereo-tactically injected with 7.5 × 104 GL261 cells: a median incision of ~1 cm was made; a burr hole was drilled in the skull and cells were injected in the right striatum (−2 mm lateral and +1 mm anteroposterior from the bregma). A total cell suspension of 4 µL in sterile PBS was injected with a Hamilton syringe at a rate of 1 µL/min at 3 mm depth.

### Tumor volume evaluation and immunofluorescence on brain slices

Twenty-one days after tumor cell inoculation, glioma-bearing mice were sacrificed and brains were isolated, fixed in 4% buffered PFA and snap frozen. Coronal brain cryosections (20 μm of thickness) were prepared by standard procedures and collected every 100 μm. Tumor volume was evaluated with hematoxylin–eosin staining, as detailed by the manufacturer. Briefly, after staining, brain slices were analyzed by the Image Tool 3.0 software (University of Texas, Health Science Center, San Antonio, TX, USA). Tumor volume was calculated according to the formula (volume = t × ΣA, where t = thickness and A = tumor area/slice). Parallel cryosections were used for immunofluorescence staining of TfR1, GFAP and Ki-67. Briefly, sections were incubated with 3% goat serum in 0.3% Triton X-100 for 1 h at RT, and overnight at 4 °C with anti-TfR1 (Millipore, 1:500) and GFAP (Novus Biologicals,1:1000), anti-Ki-67 (Abcam, 1:50) in 1% goat serum, 0.1% Triton X-100. Sections were then stained with fluorophore-conjugated secondary antibodies and Hoechst for nuclei visualization.

### Immunofluorescence on cells

The immunofluorescence staining was performed on 5 × 10^4^ cells seeded on 12 mm coverslips and fixed with 4% PFA solution Then, cells were permeabilized with 0.2% triton in PBS and blocked with BSA 1% in PBS for 45 min at RT. Cells were then incubated with primary antibodies overnight (γH2A.X, Cell Signaling, 1:1000; CD71, Thermo-Fisher, 1:100) in 0,1% of BSA-PBS. Cells were then stained with fluorophore-conjugated secondary Abs and Hoechst for nuclei visualization.

### Survival analysis

For survival analysis glioma injected mice were daily monitored following cell transplantation. The endpoints were determined by the Humane Endpoint table. The mean survival time was calculated using the Kaplan–Meier method and statistical analysis was performed using a Log-rank test.

### Samples extraction

Brain tissue was accurately weighted and then homogenized in 400 µL 20 mM Tris buffer at pH 7.4, +800 µL 1% formic acid in acetonitrile, sonicated, and centrifuged to eliminate debris. Plasma was treated similarly. The supernatant was deproteinized and delipidized by using the Ostro plates as per manufacturer’s instructions (Waters, Milford, MA, USA).

### Ultra-high performance liquid chromatography and mass spectrometry

Chromatographic analyses were performed on a Waters Acquity H-Class UPLC system (Waters, Milford, MA, USA), equipped with a quaternary solvent manager, a sample manager with a flow through needle system, a photodiode array detector and a single-quadruple mass detector with electrospray ionization source (ACQUITY QDa).

The column was a reverse phase C18 Kinetex (100 mm × 3.2 mm i.d., 2.6 µm particle size, 100 Å) (Phenomenex, Torrance, CA, USA). The mobile phase was: solvent A 0.1% formic acid in water, solvent B was 0.1% formic acid in acetonitrile. The flow rate was 0.5 mL/min, the column temperature was set at 35 °C and the elution was performed with the following gradient: 2%B for 1 min, then 48% B in 7 min, followed by a wash with 100%B and a re-equilibration of the column for 4 min. Samples were diluted in the mobile phase and injected through the needle. The PDA detector was set up in the range 210–600 nm. Mass spectrometric detection was performed in the positive electrospray ionization mode, using nitrogen as the nebulizer gas. Analyses were performed in the Total Ion Current (TIC) mode with a mass range of 100–1200 m/z. The capillary voltage was 0.8 kV, cone voltage 8 V, ion source temperature 120 °C and probe temperature 600 °C. In these chromatographic conditions Genz-644282 has a retention time of 4.7 min, a 408.06 m/z for the MH+ form, 204.5 m/z for the MH2+ form, and λmax at 298.8 and 271.4 nm in the UV–Vis region. Quantification of Genz-644282 compound was performed by using a standard calibration curve in the range of 0.04–10 µM. The calibration curve was obtained by plotting the peak-area ratio of Genz-644282 analyte versus its concentration (*R*^2^ = 0.9997).

## Results

### HFt affinity for human and murine TfR1

**Fig. 2:**
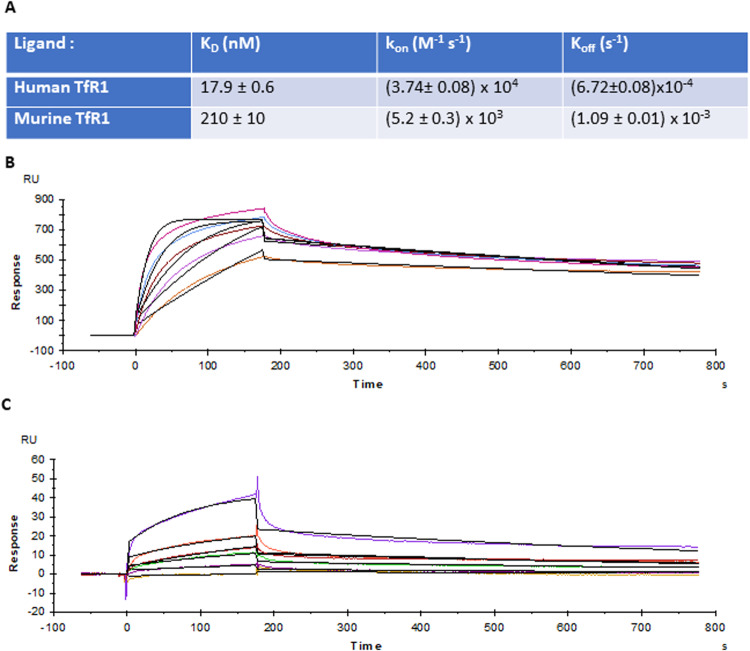
SPR experiments. **A** Thermodynamic and kinetic parameters of the interaction of HFt (analyte) with human and murine TfR1 (ligands). **B** Sensorgrams showing the interaction of HFt with human TfR1 immobilized onto a HisTag sensorchip. Sensorgrams show HFt injection (time 0–180 s) at 30 µL/min at the following concentrations: 2, 1, 0.5, 0.25, 0.125 µM (purple, blue, black, red, green sensorgrams, respectively), followed by buffer injections (180–780 s) to monitor analyte dissociation. All curves were fitted as single 1 :1 interactions; fittings are shown as black curves. **C** Sensorgrams showing the interaction of HFt with murine TfR1 immobilized onto a HisTag sensorchip. Sensorgrams show HFt injection (time 0–180 s) at 30 µL/min at the following concentrations: 2, 1, 0.5, 0.25, 0.125 µM (purple, blue, black, red, green sensorgrams, respectively), followed by buffer injections (180–780 s) to monitor analyte dissociation. All curves were fitted as single 1:1 interactions; fittings are shown as black curves.

The-0504 is based on a modified, recombinant human ferritin molecule, named The-05, that instead of iron contains about 70–80 Genz-644282 molecules entrapped in its hollow cavity [38]. The-05 was designed to be activated by metalloprotease-dependent cleavage in tumor microenvironment and transformed into HFt, that has high affinity for human TfR1. Surface Plasmon Resonance (SPR) experiments were carried out to assess the thermodynamical and kinetic parameters of the interaction between HFt (analyte) and human or murine TfR1 (ligands) in a Biacore X-100 apparatus (Fig. 2). The experiments show that HFt is able to bind to both ligands in the nanomolar range. However, HFt showed a reduced binding to the murine TfR1 receptor by sharply increasing the dissociation constant (Kd) from 18 nM to 210 nM for human and murine receptors, respectively. This may reduce the activity of The-0504 for murine glioma with respect to human glioma.

### The-0504 kills glioma cells

**Fig. 3:**
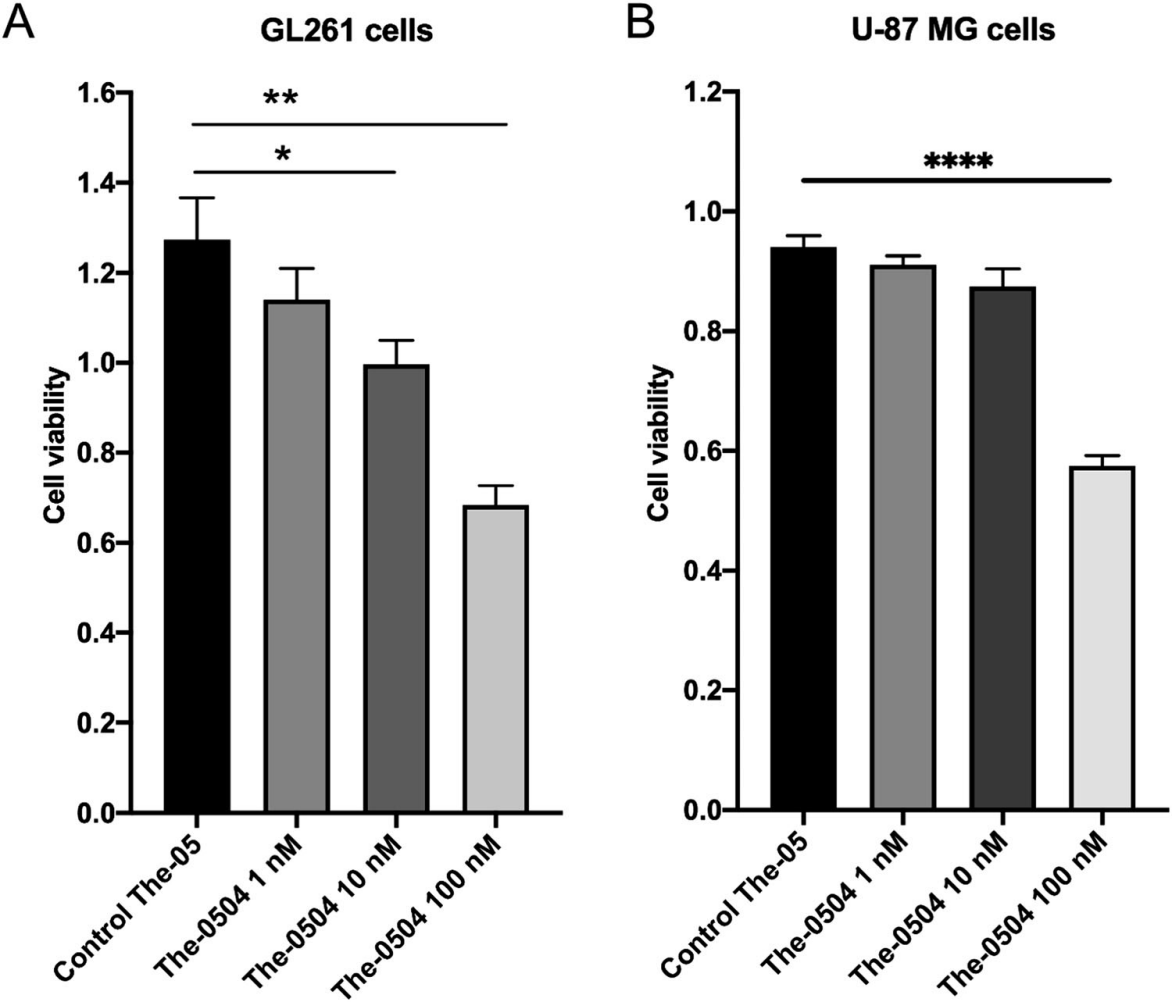
The-0504 kills murine and human glioma cells. **A** GL261 murine glioma cells and **B** U-87MG human glioblastoma cells (1 × 10^4^) in DMEM 10% FBS, were cultured in 96 multiwell plates. Cell viability of glioma cells exposed to different concentrations of The-0504, expressed as Genz-644282 concentrations (1–10–100 nM), for 48 h and determined by MTT assay (*n* = 6 well per condition). Error bars indicate means ± SEM. *p < 0.05 and **p < 0.01 as determined All Pairwise Multiple Comparison Procedures ANOVA (Holm–Sidak method). ****p < 0.0001 as determined All Pairwise Multiple Comparison Procedures ANOVA (Tukey method).

In order to investigate whether the potent The-0504 nanocarrier was effective also against glioma cells, murine GL261 and human U-87 MG cells (1 × 10^4^) were cultured in 96 multiwell plates, and exposed to different concentrations of The-0504 (1–10–100 nM) to assess the viability of cells. As shown in Fig. 3, treatment with The-0504 decreases cell viability with respect to control (empty The-05 treatment) in a concentration-dependent fashion. At 48 h after treatment, the IC50 is about 100 nM (*P* < 0.01 for GL261 cells; *P* < 0.0001 for U-87 GL cells), indicating that the drug is able to significantly reduce the proliferation of tumor cells in these two glioma models, both expressing high level of TfR1 receptor (Supplementary Fig. 1).

**Fig. 4:**
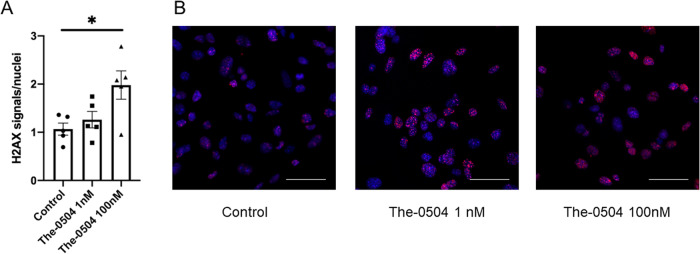
Dose-response of *γ*H2AX foci formation in GL261 cells after The-0504 treatment. **A** *γ*H2AX foci were quantified as foci signal per nucleus for each dose in controls and THE 0504 treated cells at different concentrations for 6 hours. **B** Representative immunofluorescence staining of GL261 glioma cells treated with vehicle or different The-0504 concentration for 6 h. Nuclei were stained in blue with Hoechst and foci in red with anti *γ*H2A.X antibody. Scale bars 50 µm.

To investigate the mechanism of action of the The-0504 nanocarrier, we carried out experiments to test DNA damage in vitro, in order to assess whether the Topo 1 inhibitor Genz-644282, delivered as The-0504 complex into the nucleus, may induce DNA double-strand breaks (DSBs). DSBs are lethal DNA injuries that can be caused by cytotoxic chemical agents as well as environmental and physical damage. They were assessed by staining cells with an antibody against histone H2AX (S139 phosphorylation), that is a specific DSBs marker [43]. GL261 glioma cells were thus treated with 1 or 100 nM Genz-644282 as The-0504. Immunofluorescence staining revealed that after 6 h of treatment with The-0504, foci expressing the DNA double-strand break marker γH2AX were induced in GL261 cells, since γH2AX expression increases up to 2-fold as compared to control (Fig. 4).

### Glioma cells contains high levels of TfR1

**Fig. 5:**
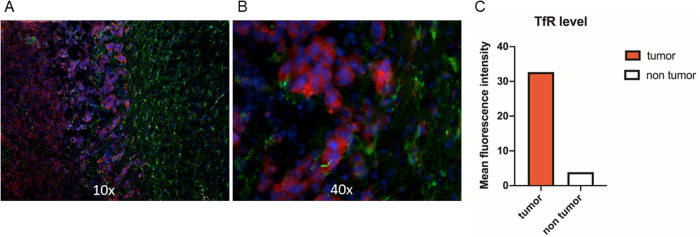
The ferritin/transferrin receptor TfR1 is expressed at high levels in tumor tissue. Immunofluorescence experiment on C57BL6/N mouse bearing GL261 glioma three weeks after cell inoculation: in vivo expression of TfR1 (transferrin/ferritin receptor, red) and GFAP (glial fibrillary acidic protein, green); nuclei (Hoechst) are in blue; **A** ×10 magnification; **B** ×40 magnification. **C** mean fluorescence intensity of TfR1 (red channel) in glioma cells (red) and mouse brain cells (white).

To evaluate the relative expression of the ferritin receptor TfR1 in vivo, an orthotopic model of murine glioma was built (see Materials and Methods section). Twenty-one days after GL261 glioma cells inoculation, glioma-bearing mice were sacrificed, brains were isolated, fixed and frozen, and coronal brain cryosections were prepared for immunofluorescence staining of ferritin receptor TfR1. Sections stained with fluorophore-conjugated secondary antibody and Hoechst for nuclei visualization (Fig. 5) showed that TfR1 is expressed at high levels in the glioma cells, while its expression is low in the peritumoral and healthy brain parenchyma. Measurement of the mean fluorescence intensity (MFI) of TfR1 in glioma cells compared to mouse brain cells shows that TfR1 is at least 8 times more expressed in the tumor tissue than in the surrounding region. Possibly, this ratio could even higher, as some tumor cells may have spread outside the primary tumor site.

These results demonstrate a selective overexpression of TfR1 in glioma cells and prompt the use of The-05 protein for the selective delivery to tumor cells of the non-camptothecin topoisomerase I inhibitor, Genz-644282.

### Efficient and selective nose-to-brain in vivo delivery of Genz-644282 to GL261 glioma cells

In order to assess the ability of The-0504 to selectively target to glioma tumor, we administered it *via* the nose-to-brain route and quantified the drug Genz-644282 by Ultra-High Performance Liquid Chromatography, both in the brain and in the blood.

**Fig. 6:**
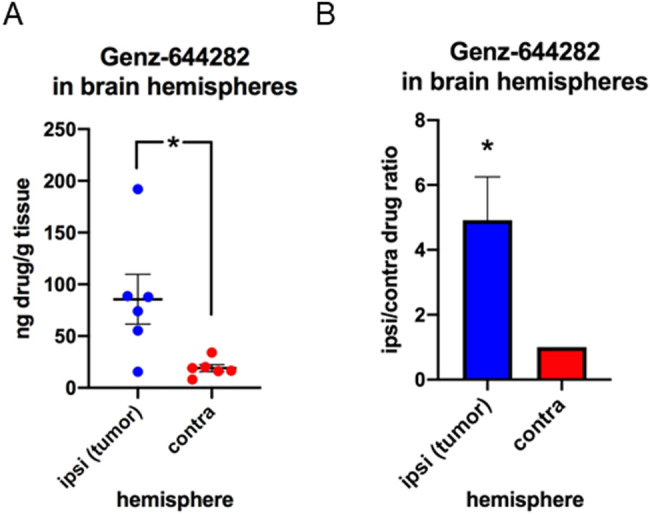
Drug content in tumoral and contralateral hemispheres after brain-to-nose administration of The-0504. Quantification of Genz-644282 by UPLC in tumor (ipsi) brain hemisphere and contralateral (contra) hemisphere 1 h after brain-to-nose administration of 15 µL The-0504 (0.9 mg/kg) in C57BL6/N mouse bearing GL261 glioma, three weeks after cell inoculation (n = 6 mice). **A** overall quantification of ng Genz-644282/g tissue in the brain hemispheres of 6 mice; the values are indicated for each brain hemisphere (blue: tumor hemisphere; red: contralateral hemisphere); average and SEM are indicated for each analysis (unpaired t test; *P < 0.05). **B** quantification of pairwise tumor/contra hemisphere drug ratio (ng Genz-644282/g tissue) for each experiment (ratio paired t test; *: P < 0.05).

Since glioma cells were inoculated in the right striatum, quantification of Genz-644282 was assessed in tumor (ipsi) brain right hemisphere and in contralateral (contra) left hemisphere, 1 h after brain-to-nose administration of 15 µl of The-0504 in C57BL6/N mouse bearing GL261 glioma (0.9 mg/kg), three weeks after tumor cell inoculation (Fig. 6). Lyophilized The-0504 powder was dissolved in sterile water at a concentration of about 3.8 mM (expressed in Genz-644282, i.e., 48 µM in ferritin). Genz-644282 is soluble at these high concentrations only in 100% DMSO, while it is not soluble in aqueous solutions. Obviously, these conditions are not suitable for a brain-to-nose administration, excluding the possibility of using non-HFt-encapsulated Genz-644282 in control experiments.

The quantification of ng Genz-644282 per g of tissue in the brain hemispheres of 6 treated mice showed that the right, glioma-containing brain hemisphere (blue) contains an average of almost 5 times higher drug amount with respect to the left, non-tumor inoculated brain hemisphere (red) (unpaired t test; *P = 0.03; ratio paired t test; *P = 0.027). These data point-out to a high specificity for the TfR1-mediated delivery of The-0504 compound. Thus, the drug Genz-644282 can be efficiently and selectively delivered to TfR1-expressing glioma cells by the The-05 nanovector, that is able to maintain the drug itself soluble in aqueous solution and to cross the blood-to-brain barrier.

We also performed the assessment of the amount of the chemotherapeutic drug Genz-644282 in mice plasma, 15′, 30′, 1 h, 2 h and 4 h after intranasal administration of 15 µl of The-0504 (0.9 mg/kg; 22 µg total Genz-644282 drug). These experiments (Supplementary Fig. 2) showed that negligible amounts of drug (below 11 ng/g blood) are present at the times studied in animal plasma upon intranasal treatment, suggesting that the therapeutic efficacy of nose-to-brain administration to mice does not depends on losses by nose-to-blood routes such as the nose-esophagus-stomach route. This further demonstrated that the intranasal administration of The-05 is safe and well tolerated. Nevertheless, a complete toxicological study must be carried-out in the future to apply this novel route of administration to The-0504.

### Nose-to-brain administration of The-0504 reduces glioma volume and cell proliferation in the animal model

To assess the therapeutic activity of The-0504 also in vivo, the same murine model was used. C57BL6/N mice bearing GL261 glioma were sacrificed after six intranasal administrations and evaluated for the relative presence of residual tumor. Mice were treated bi-weekly with The-0504 or with empty The-05 as control, starting one week after tumor inoculation.

**Fig. 7:**
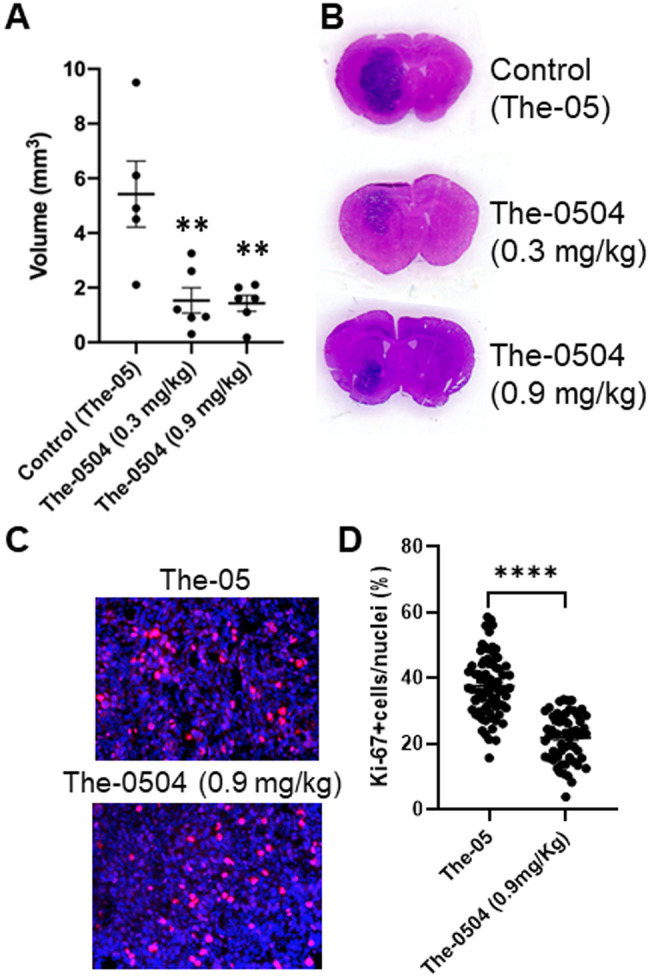
Tumor volume after intranasal administration of The-0504 and proliferation analysis. **A** Tumor volume after 6 nose-to-brain administrations (2/week) of 15 µL empty The-05, 5 µL The-0504, or 15 µL The-0504 in C57BL6/N mouse bearing GL261 glioma (0.3 and 0.9 mg/kg, respectively), starting one week after tumor cell injection. Single experiments, mean ± SEM. **P < 0.01 (N = 5–6), as determined by All Pairwise Multiple Comparison Procedures ANOVA (Holm–Sidak method). **B** Representative images of tumor size in mice brain coronal slices stained with Hematoxylin & Eosin for all groups. **C** Representative fields of view (×20 magnification) of The-05 and The-0504 treated tumors in coronal brain slices analyzed for Ki-67 staining (red). Nuclei were counterstained with Hoechst (blue). **D** Number of Ki-67 positive cells normalized to nuclei per field of view (FOV), expressed as a percentage for the two groups. n = 3 mice per group/22–24 slices/66–72 FOVs; data are expressed mean ± SEM, ****P < 0.0001 by unpaired t-test.

After six nose-to-brain administrations of 15 µl empty The-05, the mean tumor volume was 5.4 ± 1.2 mm^3^. In contrast, after six treatments with 5 µl or 15 µl of The-0504 (0.3 mg/kg and 0.9 mg/kg, respectively), the mean tumor volume was 1.5 ± 0.5 mm^3^ (*P* < 0.01) and 1.4 ± 0.3 mm^3^ (*P* < 0.01) respectively (Fig. 7A, B). In addition, we evaluated tumor cell proliferation by immunofluorescence analysis of the Ki-67 marker on coronal glioma-bearing sections (Fig. 7C). As shown, the percentage of Ki-67 positive cells was significantly reduced following treatment with The-0504 (0.9 mg/kg) compared to the control (Fig. 7D), supporting the observed reduction in tumor volume. Therefore, these results demonstrate that nose-to-brain administrations of The-0504 are able to reduce glioma volumes affecting tumor cell proliferation in animal models in a statistically significant manner. This is remarkable also considering that murine glioma cells display a TfR1 receptor characterized by a lower binding affinity to the human ferritin in comparison to the human counterpart (Fig. 2A).

### Nose-to-brain administration of The-0504 increases the survival of glioma-bearing animals

**Fig. 8:**
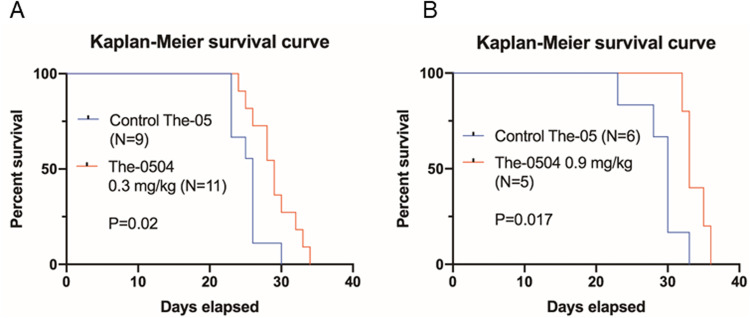
Survival analysis of glioma mouse model after brain-to-nose administration of The-0504. Survival of C57BL6/N mouse bearing GL261 glioma after 6 brain-to-nose administrations (2/week) of The-0504, starting one week after inoculation. **A** intranasal 5 µL/treatment The-0504 (0.3 mg/kg, N = 11 mice), vs. intranasal 5 µL PBS/treatment (control; N = 9 mice); *P* = 0.02, by log rank statistic. **B** intranasal 15 µL /treatment The-0504 (0.9 mg/kg, N = 5 mice), vs. intranasal 15 µL PBS/treatment (control; N = 6 mice); *P* = 0.017, by log rank statistic.

Survival of glioma-bearing animal models was assessed in two additional in vivo experiments. The same dose regimen of the previous experiment was used, that is six brain-to-nose administrations (2/week) of The-0504, starting one week after inoculation (Fig. 8).

After six nose-to-brain administrations of 5 µl The-0504 (0.3 mg/kg, N = 11 mice), the mean overall survival was 29 days, vs. 26 days upon 6 nose-to-brain administrations of 5 µl PBS/treatment (control; N = 9 mice) (Fig. 8A; *P* = 0.02). In a second experiment, carried out subsequently, after six nose-to-brain administrations of 15 µl The-0504 (0.9 mg/kg, N = 5 mice) or of 15 µl PBS/treatment (control; N = 6 mice), the mean overall survival was 33.8 ± 0.7 days and 29.0 ± 1.3 days respectively (Fig. 8B; *P* = 0.017). The experiment shows that nose-to-brain administration of The-0504 increases the survival of the glioma animal model in a statistically significant manner.

### Intravenous administration of The-0504 reduces glioma volume in the animal model

**Fig. 9:**
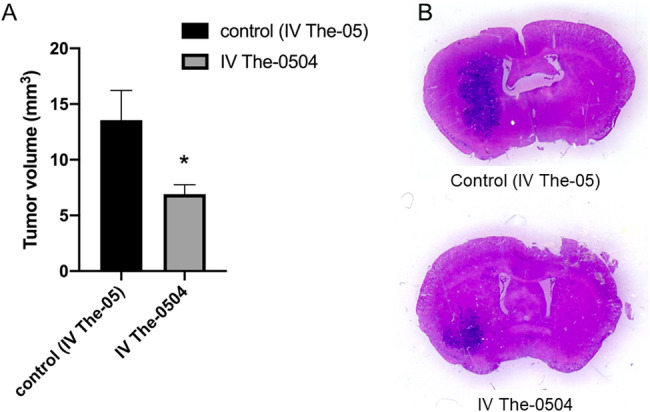
Tumor volume after IV administration of The-0504. **A** Tumor volume after 6 IV administrations (2/week) of 200 µL empty control The-05 and of 200 µL The-0504 (2.0 mg/kg) in C57BL6/N mouse bearing GL261 glioma, starting one week after inoculation. Single experiment, Mean ± SEM. *: P < 0.05 (N = 6), unpaired t test. **B** Representative images of brain coronal slices with glioma stained with Hematoxylin & Eosin in control and The-0504 treated mice.

To assess whether also an intravenous (IV) administration of The-0504 is able to reduce glioma volume in the animal model, an additional experiment was carried-out. C57BL6/N mice bearing GL261 glioma were sacrificed after six IV administrations and evaluated for the presence of residual tumor. Mice were treated bi-weekly with The-0504 or with empty The-05 as control, starting one week after tumor inoculation (Fig. 9).

After six IV administrations of 200 µL empty The-05 or with 200 µL of The-0504 (2.0 mg/kg), the mean tumor volume was 13.4 ± 2.6 mm^3^ and 7.1 ± 0.7 mm^3^ (*P* < 0.05).

Intravenous administration of The-0504 therefore also reduces glioma volume in the animal model in a statistically significant manner. However, the tumor volume is reduced by 53% as an average after 6 IV treatments with 2.0 mg/kg The-0504, while the tumor volume is reduced more than 70% upon nose-to-brain treatment carried out with 0.3 mg/kg The-0504 (Fig. 7), indicating the intranasal route as the most effective one, using about 7 times less drug.

## Discussion

Gliomas are one of the most serious threats to human health, and therapeutic options are very limited, especially for high-grade gliomas, which are very aggressive. In addition, the BBB prevents most chemotherapy drugs from penetrating the brain. Nanocarriers are potentially valuable for glioma targeting. HFt-based approaches are highly sought-after because ferritin is non-immunogenic, biodegradable, highly soluble, and capable of protecting the payload from external agents. Furthermore, HFt is among the molecules that can cross the BBB. However, only a few studies have used ferritin-based approaches against glioma, such as HFt loaded with doxorubicin and paclitaxel [24, 35–37].

Previously, we modified the HFt surface to increase solubility and tumor specificity. This modified protein, named The-05, was able to encapsulate high amounts of the potent non-camptothecin topoisomerase I inhibitor Genz-644282. The The-05/Genz-644282 complex, named The-0504, was already demonstrated to be effective against a panel of different cancer cells both in vitro and in vivo [38, 40].

In the current study, we administered the loaded nanovectors to a syngeneic model of murine glioma, both intravenously and *via* a nose-to-brain route, to evaluate its potential in high-grade glioma, which expresses high amounts of the ferritin transporter, TfR1 [44]. A similar intranasal route has been used by two other groups for the delivery of HFt-derived nanoparticles as a possible vaccine for influenza or as a possible drug against lung cancer [45, 46]. In addition, nose-to-brain administration of three other types of nanosystems was attempted with promising results for the treatment of gliomas, using mixed micelles consisting of Tat-conjugated polymer micelles and stearoyl-modified bombesin, self-assembled nanoparticles of therapeutic RAGE-antagonist peptides and anti-miR21 antagomirs, and anti-oligonucleotide co-micelles against miR-21 [47–49].

Here we demonstrate that The-0504 enables efficient and selective drug delivery to glioma cells in vivo compared to normal brain cells when administered via direct and short nose-to-brain route.

In terms of therapeutic activity, both intranasal and intravenous delivery routes have been shown to be effective in reducing glioma growth in the animal models. In particular, nose-to-brain administration of The-0504 was able to reduce glioma volume more (about 20%) and at lower dose (7 times) than the intravenous administration. Although a thorough evaluation of the biological activity against murine glioma is not the primary focus of this paper, it is noteworthy that a significant tumor reduction was observed in this model. In fact, as reported for the first time here, the affinity of HFt for human TfR1 is one order of magnitude higher than that for murine TfR1 (Fig. 2). Therefore, it is reasonable to expect a higher therapeutic response in human gliomas than in murine GL261 gliomas. This will be the subject of future studies.

In conclusion, this work is a proof-of-concept study for the use of stimuli-sensitive ferritin-based nanodrugs against gliomas. Here, we demonstrate that a route of administration with high patient compliance, such as nose-to-brain drug delivery, can be achieved using BBB-passing modified ferritin-based nanocarriers. Nose-to-brain chemotherapy delivery to gliomas via The-0504 enables selective tumor targeting and accumulation, reducing the impact of chemotherapy on normal brain cells and potentially improving prognosis. Therefore, this work could pave the way for a new, safe, and direct drug delivery method for brain diseases, especially brain tumors.

### Supplementary information

Supplemental Material

## Data Availability

The authors declare that the data supporting the findings of this study are available within the paper and its Supplementary Information files. Should any raw data files be needed in another format they are available from the corresponding author upon reasonable request.
