# Supplementary material for: Nose-to-brain selective drug delivery to glioma via ferritin-based nanovectors reduces tumor growth and improves survival rate
Source: Cell Death Dis. 2024 Apr 13;15(4):262. doi: 10.1038/s41419-024-06653-2 (PMC11016100; doi:10.1038/s41419-024-06653-2)

**Supplementary Figure 1**

**U-87MG cells express CD71**

Immunofluorescence staining for nuclei (Hoechst, blue) and CD71 (red) on human U-87MG glioblastoma cell line. Scale bar= 60 µm


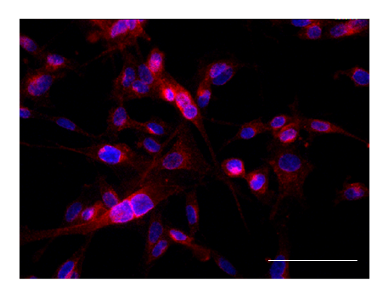


**Supplementary Figure 2**

Assessment of the amount of the chemotherapeutic drug Genz-644282 in plasma, 15’, 30’, 1h, 2h and 4h after intranasal administration of 15 µL The-0504 (0.9 mg/kg; 22 µg total Genz-644282 drug) in C57BL6/N mice. In all samples, the amount of Genz-644282 is close to the limit of quantification.


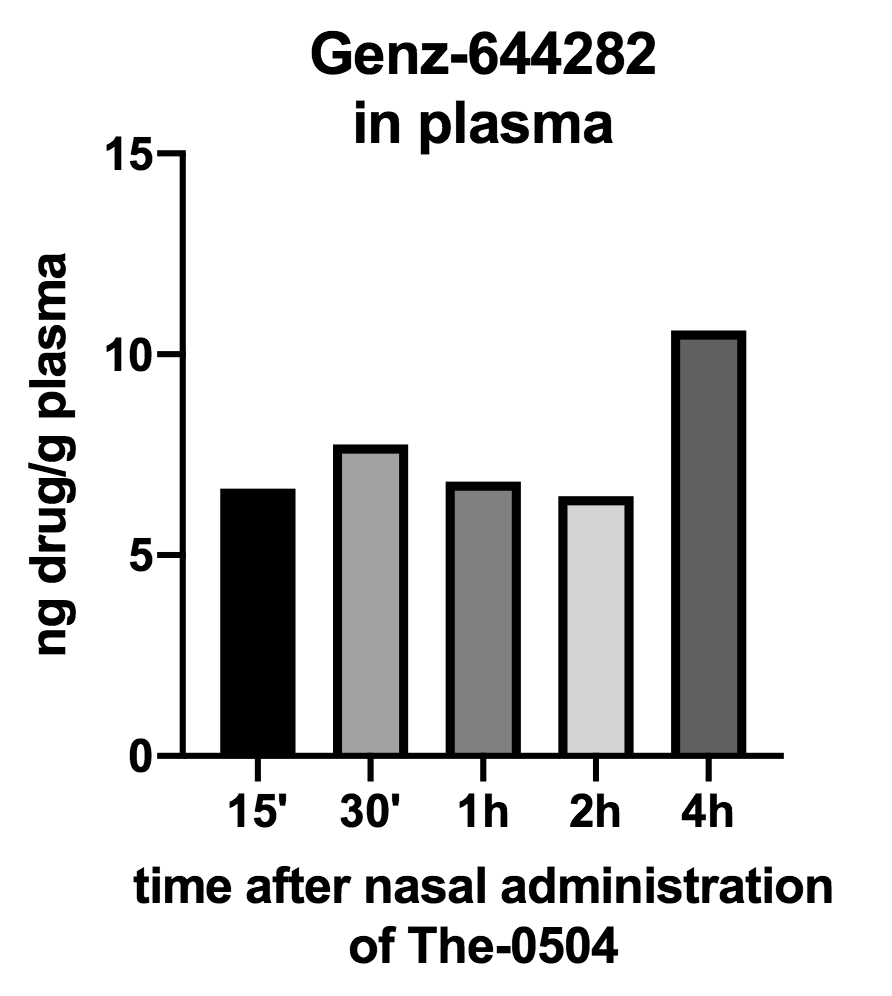

Supplement: Supplementary file 1 — Supplemental Material [file 41419_2024_6653_MOESM1_ESM.docx]
